# Evolution of opercle shape in cichlid fishes from Lake Tanganyika - adaptive trait interactions in extant and extinct species flocks

**DOI:** 10.1038/srep16909

**Published:** 2015-11-20

**Authors:** Laura A. B. Wilson, Marco Colombo, Marcelo R. Sánchez-Villagra, Walter Salzburger

**Affiliations:** 1 School of Biological, Earth and Environmental Sciences, University of New South Wales, Sydney, NSW 2052, Australia; 2 Zoological Institute, University of Basel, Vesalgasse 1, CH 4051, Basel, Switzerland; 3 Paläontologisches Institut und Museum, Karl-Schmid Strasse 4, CH 8006, Zürich, Switzerland

## Abstract

Phenotype-environment correlations and the evolution of trait interactions in adaptive radiations have been widely studied to gain insight into the dynamics underpinning rapid species diversification. In this study we explore the phenotype-environment correlation and evolution of operculum shape in cichlid fishes using an outline-based geometric morphometric approach combined with stable isotope indicators of macrohabitat and trophic niche. We then apply our method to a sample of extinct saurichthyid fishes, a highly diverse and near globally distributed group of actinopterygians occurring throughout the Triassic, to assess the utility of extant data to inform our understanding of ecomorphological evolution in extinct species flocks. A series of comparative methods were used to analyze shape data for 54 extant species of cichlids (N = 416), and 6 extinct species of saurichthyids (N = 44). Results provide evidence for a relationship between operculum shape and feeding ecology, a concentration in shape evolution towards present along with evidence for convergence in form, and significant correlation between the major axes of shape change and measures of gut length and body elongation. The operculum is one of few features that can be compared in extant and extinct groups, enabling reconstruction of phenotype-environment interactions and modes of evolutionary diversification in deep time.

Understanding how organismal diversity is generated and maintained, why some groups diversify when others remain relatively unchanged over geological time, and how organisms adapt to and interact with the environment are key challenges in evolutionary biology. Adaptive radiations, defined as rapid and extensive diversifications from an ancestral species that result in descendants adapted to exploit a wide array of ecological niches^1^,^2^, are widely recognized as fundamental subjects of investigations into organismal diversification.

The species flocks of cichlid fishes from the East African Great Lakes collectively represent an unparalleled example of adaptive radiation in vertebrates^3^,^4^,^5^,^6^. In all of the three major lakes, one or several species have radiated to produce flocks comprising more than 500 species each in Lakes Malawi and Victoria, and at least 200 species in Lake Tanganyika (LT)^7^, which is the oldest of the three with an estimated age of around nine to 12 million years^8^,^9^. Unlike the quasi-monophyletic haplochromine species flocks in Lakes Malawi and Victoria, the species flock in Tanganyika consists of several ancient lineages that radiated in parallel^9^,^10^,^11^. Molecular markers have been used to reconstruct the recent history of the LT radiation, revealing that the LT species flock was established in a series of cladogenic events that coincided with changes in the lake’s environment. An initial diversification event by seeding lineages occurred around the early stage of lake formation, represented by several shallow protolakes at around 9–12 MYA^8^,^9^. A subsequent diversification, involving seven ancient lineages and referred to as the ‘primary lacustrine radiation’, occurred around the time that the protolakes became deeper and joined to form a single deep lake, around 5–6 MYA^9^. LT cichlids are the most morphologically, ecologically and behaviorally diverse of the three lake flocks, and a number of studies have explored evolutionary patterns in the group. These include coloration patterns^12^,^13^, parental care strategies^14^,^15^,^16^, patterns of mouth morphology^17^,^18^,^19^, and brain and body size evolution^18^,^19^,^20^,^21^,^22^.

Among the morphological traits examined in LT cichlids so far, most comprise combinations of linear measurement or scored character data and, with the notable exception of body shape and size measures, few traits are directly amenable to comparison with other species flocks, such as sticklebacks and Antarctic notothenioids, which represent radiations of different geological age that have occurred in different environmental settings (marine, lacustrine, riverine). Uncovering commonalities in trait complex evolution in phylogenetically, morphologically and ecologically distinct species flocks would be highly desirable in assessing key questions underpinning how adaptive radiation progresses. Issues include the general extent to which diversification occurs in stages^23^,^24^, recently tested in LT cichlids by Muschick and colleagues^19^, and how well an early burst model, which predicts that major ecological differences occur early in a clades’ history^25^, fits adaptive radiation in fishes (see e.g.^26^). Illuminating trait patterning in deep time would be equally valuable, by focusing attention towards searching for traits that may also be measured in extinct species flocks (e.g.^25^,^27^).

In this paper, we build upon our earlier geometric morphometric investigations of operculum shape in extant^28^ and extinct species flocks^29^ by quantifying evolutionary patterns in this trait for an extensive sample of LT cichlids. The operculum is a flat and slightly curved bone plate that, together with the suboperculum, makes up the gill cover in osteichthyans (Fig. 1). It forms a ball-and-socket connection with the hyomandibula, which enables inward-outward movement of the gill cover to expand and compress the opercular chamber during the suction pump phase of the respiratory cycle^30^,^31^. In cichlids, the operculum is connected to the neurocranium (via m. levator operculi) and the gill cover complex forms a second mechanism assisting in mouth opening^32^. The considerable diversity in operculum shape and size among osteichthyans has been attributed to the important role of this bone in respiration and the jaw opening mechanism of some fishes through its functional connectivity to the lower jaw^33^. Owing to these properties, and further supported by insight from studies of operculum morphogenesis in zebrafish that have illuminated genetic pathways influencing its shape and size (e.g.^34^,^35^), the operculum has been the subject of several investigations, particularly in the threespine sticklebacks^33^,^36^,^37^. The occurrence of a parallel divergence in operculum shape following a ‘dilation-diminution model’, defined as dorsal-ventral compression coupled with anterior-posterior extension of the outline shape^33^, has been demonstrated to be a widespread phenomenon between oceanic and freshwater threespine sticklebacks^38^,^39^,^40^. Differences in operculum shape have also been found between sticklebacks inhabiting deep lakes, shallow lakes and streams, indicating a functional difference among phenotypes^37^. Recent analysis of operculum shape in Antarctic notothenioids, using phylogenetic comparative methods, revealed also a general trend in shape change along a macrohabitat-related axis (benthic-pelagic^28^), further highlighting the utility of this trait in assessing ecomorphological interactions on a broad scale. That earlier study revealed evolutionary patterns in shape best fit a model of directional selection (Ornstein-Uhlenbeck), and did not support an early burst model of adaptive radiation in notothenioids^28^. Importantly, the operculum is one of only few morphological features that can be studied in fossil groups because it is commonly well preserved.

The extent to which observed patterns in operculum shape evolution among extant species flocks may be similarly recovered in extinct species flocks requires considerable further effort to understand. Previously operculum shape evolution has been studied in a subset of the diverse species flock of *Saurichthys* (>35 species^41^), a near globally distributed genus of actinopterygian fishes that occurred from the Late Permian (245 MYA) to the Early Jurassic (176 MYA)^42^. Being the presumably first group of fishes to have evolved an elongated, slender body plan, saurichthyids have been reconstructed as bearing physical resemblance to the modern day garfish, likely a fast-swimming predator, and are known to have occupied both marine and freshwater realms^42^. Owing to their rather distinctive morphology, saurichthyids have been quite well-documented in the fossil record^41^ and particularly a number of exceptionally preserved specimens are known from the UNESCO site of Monte San Giorgio in Switzerland, allowing for detailed study of axial elongation patterns^43^,^44^. Similar to the dilation-diminution model uncovered in studies by Kimmel and colleagues, species-specific change in operculum shape among members of the genus *Saurichthys* was concentrated to a narrowing along the anterior-posterior margin antagonistically coupled with perpendicular extension along the dorsal-ventral axis^29^.

As a preliminary pathway to uniting evolutionary patterns for trait data in extinct and extant species flocks, we here place our earlier data on opercle shape and body elongation in *Sauricthys*^29^,^44^ within the framework of a much larger sample of LT cichlid data, for which we are able to measure the same traits, and complement those with additional ecological variables. The species flock of *Saurichthys* was chosen as example for this study because it possesses several favorable attributes. The saurichthyids are a distinctive group that is well-documented from Triassic deposits in Europe, particularly the Besano and Cassina formations (Monte San Giorgio, Switzerland) and the Prosanto formation (Ducan-Landwasser, Switzerland), thereby allowing for detailed palaeoecological, faunal and stratigraphical information to be extracted for numerous species (e.g.^45^), and ultimately enabling temporal changes in morphological disparity to be quantified. Previous work on the operculum of *Saurichthys*^29^,^46^,^47^ has indicated that opercle shape is a key feature for distinguishing among several species, and considerable variation in opercle shape has been linked to behavioural differences. A comprehensive examination of operculum variation and evolution in saurichthyids is of particular interest for understanding ecomorphotype segregation for sympatric groups^47^.

Our cichlid dataset contains a considerable proportion of species present in LT (Fig. 2), including the most abundant ones that coexist in the southern basin of the lake^19^, and spans the majority of LT cichlid tribes. Ecological diversity is well represented in the sample, which includes epilithic algae grazers, scale eaters, fish hunters, invertebrate pickers and species that dwell in sandy, rocky or open water areas. Using the LT cichlid data set, we apply phylogenetic comparative methods to a) examine the patterns of opercle shape and size disparity over time; b) test for phenotype-environment correlations between operculum shape and size using stable isotope data as proxy for macrohabitat and trophic niche; c) examine whether operculum shape and size are related to recognized adaptive trait complexes and assess the utility of those interactions for data from fossil species; and d) test the fit of competing macroevolutionary models to our data.

## Results

### Operculum shape and form space

Phylomorphospace plots indicated a considerable amount of variation in operculum shape and overlap between members of different tribes (Fig. 3). The first PC axis (43.9% variance) separated Bathybatini plus the lamprologine *A. calvus* (Fig. 3A (i)) from the other groups. Positive PC1 scores, exhibited by members of Bathybatini, reflected compression along the anterior dorsal and posterior ventral margin of the operculum along with extension along the posterior dorsal and anterior ventral margin (Fig. 3A). Negative scores along PC2 (20.4%) reflected a widening of the operculum along the anterior-posterior axis and a shortening along the dorsal-ventral axis, whereas positive scores reflected the reverse. Generally, some separation along this axis is evident between Lamprologini (negative scores) and Tropheini (positive scores), overlapping with Ectodini, as for example the second most extreme positive value is represented by a member of the latter tribe (*O. ventralis*: ophven). Noteworthy is that the PC1-PC2 plot (Fig. 3A) shows more or less complete overlap in morphospace occupation for Tropheini and Ectodini relative to different regions of morphospace occupied by Lamprologini and Bathybatini. A division in morphospace occupation is also visible for members of Lamprologini wherein members of *Neolamprologus* have negative scores along PC2 and are separated from other species in Lamprologini, belonging to *Lamprologus*, *Altolamprologus* and *Lepidolamprologus*. PC3 (12.5%) largely reflected shape changes occurring along the dorsal edge of the bone, resulting in a more asymmetrical shape of the bone at the dorsal margin, either angled with more bone extending on the anterior (negative PC3 score) or posterior (positive PC3 score) side (Fig. 3B). PC3 resulted in some minor separation between Tropheini and Ectodini (slightly higher PC3 scores).

The projection of shape data into form space, including centroid size, resulted in two PC axes comprising more than 95% of the sample variance. PC1 contains size-related shape change, accounting for 92.4% of sample variance. This reflects a change from species with smaller bones exhibiting expansion and compression along the posterior and anterior margin of the operculum (e.g. *Altolamprologus compressiceps* [altcom], *Lamprologus ornatipinnis* [lamorn] and *A. fasciatus* [altfas]), respectively, to species with larger bones showing expansion of the dorsal anterior margin and compression of the dorsal posterior margin (e.g. *Bathybates vittatus* [batvit], *B. graueri* [batgra], and *Benthrochromis tricoti* [bentri]) (Supplementary Fig. 1).

### Operculum shape patterns associated with feeding ecology

CVA for feeding preference groupings resulted in a clear separation along CV1 (43.5%) between microvertebrate/algae eaters (negative scores) and piscivores (positive scores), with generalist and benthic invertebrate feeders occupying an intermediate position between the two. CV1 largely reflected shape change along the posterior margin of the operculum, with piscivores having a dorsally-broader bone that tapers postero-ventrally. CV2 (18.0%) separates the scale eaters (*Perissodus*) and zooplankton feeders, both of which are located at the positive end of CV2, from piscivores and generalist feeders, which both have negative scores for CV2 (Fig. 4A). CV2 shows that *Perissodus* and zooplankton feeders typically have a more anteriorly widened bone compared to species occupying the negative end of that axis.

CVA conducted on feeding mode revealed a clear separation between all feeding mode categories with the exception of benthic invertebrate pickers and suction groups that overlapped almost completely in CV1-CV2 morphospace (Fig. 4B). CV1 (34.9%) separated the rockpicking group (negative scores) from the scale group (positive scores), with other groups distributed in between those two extremal categories. Similar to the CVA of feeding preference, CV1 distinguished *Perissodus* as having opercles that were widened along the dorsal anterior-posterior margin. CV2 (26.7%) clearly separated the ram and suction feeders, which are shown to have a dorsally flattened opercle margin and general wider bone, from species that sandpick, algaepick and rockpick (Fig. 4B). Results of Procrustes ANOVA indicated a significant effect of both feeding mode (F_5,410_ = 3.70, *P* < 0.001) and feeding preference on operculum size and shape (F_5,410_ = 6.49–10.63, *P* < 0.001).

### Correlation between operculum shape and ecological trait and niche data

Correlations were computed using phylogenetically corrected regressions for operculum shape and form space axes, and centroid size against isotope values, gut length, gill raker traits and ER. Overall, significant results were limited to a subset of the investigated variables, with no significant relationship between operculum size or shape and gill raker numbers (grnDa, grnVa) or values of δ_13_C. Gut length data were found to be significantly correlated with PC2 (*P* = 0.003, correlation = −0.16; Fig. 5B), mainly reflecting a distinction between members of Lamproglogini, having low scores along PC2 and shorter intestine length relative to body length, and Tropheini, typically possessing longer relative intestine lengths and positive scores along PC2. A significant relationship was also found between GLTL and PC1 of form space (*P* = 0.017, correlation = 0.016), and centroid size (*P* = 0.022, correlation = −0.16; Fig. 4C). PC2 and ER were also found to be correlated (*P* = 0.041, correlation = −0.54; Fig. 5D) with more elongate species (greater values for ER) having generally more negative PC2 scores. We also examined plots of the relationship between operculum shape and ER for the six saurichthyid species. Both PC1 (*P* = 0.06, correlation = 0.56) and PC2 (*P* = 0.32, correlation = −0.26) were correlated with ER, however these correlations were not significant. The sauricthyids were more elongate (ER values of < 2) than the LT cichlids studied here, and PC1 (shown in Fig. 5E) for the saurichthyid data set expressed the same mode of shape change captured by PC2 of the LT cichlids (see shape models bordering PC2 on Fig. 3A). Among the fossil taxa, PC1 variance showed some phylogenetic grouping (Supplementary Fig. 2) and is likely explained by the large variance in body size in the sample. Apart from the small-bodied *Saurichthys striolatus* (100–180 mm^48^) (Fig. 5E), the sample included several large-bodied species (e.g. *S. costasquamosus,* >1 meter^41^). The relationship between ER and size-related shape change of the operculum requires a thorough examination in other extinct species flock. Our results recover a common pattern of size-related shape change for the two species flocks, holding promise for future examination of macroevolutionary dynamics for this trait.

### Macroevolutionary model test of operculum shape and size evolution

Model fitting results indicated that PC axes of operculum shape showed best fit to different models. In contrast to the other shape variables, PC1 was best fit by the WN model, however AICc values showed very small magnitudes of difference between that model and all others (∆AICc = 0.26–0.49), apart from BM, which was least favored (∆AICc = 5.20) (Table 1). Pagel’s λ was marginally best supported for PC2 and both BM and WN fit least well (∆AICc = 4.97–4.99) (Table 1). Again, differences were quite small for AICc values among OU, EB, Pagel’s δ and Pagel’s λ indicating a single model could not be clearly distinguished as best fit. PC3 and centroid size fit best to Pagel’s δ and, of the three shape axes examined, PC3 showed the most difference in fit across the tested models (Table 1).

Blomberg’s *K* values were less than 1 for all examined axes of shape space, and for centroid size (Table 2). Values of <1 for the *K* statistic indicate less phylogenetic signal than expected under a Brownian motion, whereas values of >1 would indicate close relatives are more similar in operculum traits than expected given the topology and branch lengths. Values ranged from *K* of 0.34 (PC1) to 0.59 (centroid size). Generally, values of *K* were quite low for PC axes, and lowest for PC1, reflected also in the low Akaike weight (probability 0.02) of that model. Our reported range of 0.34 (PC1) to 0.42 (PC2) for *K* corresponds well with that of earlier reported values for PC axes of body shape (range = 0.41–0.44), and is lower than that for PC axes of lower pharyngeal jaw (LPJ) shape (range = 0.48–0.67) (Table S3^19^). Pagel’s λ values for quantifying phylogenetic signal in the data indicate a continual increase from λ = 0.75 for PC1 to λ = 0.95 for PC3 (Table 2). Values of λ range from 0, reflecting a star phylogeny and no phylogenetic signal, to 1, which recovers the Brownian motion model. Since a Pagel’s λ value of 1 would recover the BM model, the latter result is also reflected in the substantially larger Akaike weight for the BM model fit to PC3 (probability 0.13), than the other axes (<0.03). Previous quantification of phylogenetic signal in shape data recovered similar Pagel’s λ values, ranging from λ = 0.44–0.88 for body shape and λ = 0.83–0.95 for LPJ shape^19^.

Values for α from EB model fitting were positive for all shape axes and centroid size, indicating acceleration in trait evolution. Pagel’s δ values were also greater than 1 for all measured axes and centroid size (range 5.06–8.67); δ values >1 indicate a concentration of evolution in the operculum shape and size traits towards present. If Pagel’s δ values were <1 this would indicate that branch lengths are transformed to become increasingly shorter towards the tips, meaning that trait change occurred mainly along basal branches. Operculum shape appears to be evolving more rapidly than size, as indicated by larger values for α. The time-dependent models were not better fit than alternative tested models in the case of centroid size. In contrast, for PC1 and PC2, Pagel’s δ, EB and OU appear to be considerably better supported (∆AICc >4) then the BM model (Table 1).

Pairwise distance-contrast plots indicate a general trend that is compatible with convergence, showing most species pairs occupy the quadrant of the plot represented by small morphological distances yet large phylogenetic distance (Fig. 6). Similarly, for a considerable number of species pair comparisons the observed interspecific similarity is slightly greater than expected under BM given the phylogenetic distance between the species pair (Fig. 6B). Results of pairwise comparison between the observed data and simulated data resulted in 87 species pairs being more similar than expected under BM, which is around three times more than expected by chance from the model. These pairs include a number of comparisons between members of Limnochromini and Lamproglogini.

### Disparity through time

Disparity through time analyses resulted in generally similar patterns of average clade disparities for shape and size across the time slices plotted (Fig 7). Shape disparity remained relatively stable through time (Fig. 7A), whereas size disparity (Fig. 7B) tended to decline over time until reaching a plateau around 0.65 relative time, followed by a subsequent increase. Both shape and size disparity deviated positively from simulations under BM, indicating a slightly greater amount of overlap in morphospace among sublades than would be expected under neutral evolution. Values for MDI were deviated significantly from BM simulations for both shape (*P* = 0.0049) and size (*P* < 0.001). The morphological disparity index (MDI), which indicates the amount of difference in disparity between observed trait data and data expected under BM, was greater for operculum size (MDI = 0.35) than for shape data (MDI = 0.15). Neither plots show clear evidence for an Early Burst in these traits, which is in accordance with the above reported α values for the EB calculations and the Pagel’s δ values, which together point towards more evolutionary change in the recent fauna for operculum size and shape.

## Discussion

The species flock of LT cichlids is well-recognized as an ideal model system for studying how organismal diversity emerges^5^,^49^,^50^. The operculum, a functionally-important craniofacial element for which comparative data are available from other extant species flocks and may be acquired from extinct species flocks, is here studied in a comprehensive sample of LT cichlids. Our results indicate (a) a similar mode of operculum shape change to that previously uncovered for other species flocks; (b) stability in the patterns of shape disparity through time, whereas size disparity tended to decline followed by a subsequent increase around the time of the “primary lacustrine radiation”^10^; (c) a lack of unequivocal support for a single evolutionary model, yet suggested that operculum shape evolution fit well to time-dependent models (Pagel’s δ); (d) evidence for differences in operculum shape relating to feeding preference and feeding mode, especially between piscivores and algivores, providing preliminary support for the potential utility of this trait in dietary inference; and (e) a significant relationship between operculum shape and several traits, including measures of elongation, which may also be potentially recovered for extinct species flocks.

### Relationship between operculum shape and feeding ecology

It has recently been shown that evolutionary shape change between anadromous and lacustrine sticklebacks reflects the pattern of morphological development of the opercle, namely a broadening of the anterior-posterior axis of the operculum coupled with a narrowing of the dorsal-ventral axis in freshwater sticklebacks^40^, which was further confirmed in other populations^37^. This main mode of shape change is reflected among the cichlids sampled here along PC2, which is found to correlate significantly with body elongation (ER) as well as standardized measures of gut length (GLTL). Together, the results for GLTL and δ_15_N point towards support for a relationship between operculum shape and feeding, which is shown by the results of the Procrustes ANOVAs and CVAs using feeding mode and preference (Fig. 4), and further suggested by the correlation of operculum traits with gill raker length, an additional trait that is connected to feeding, particularly processing of food items in the buccal cavity. A benthic-limnetic trend is evident in the results of the CVA based on dietary groupings, and the main axis that results in discrimination between algivore and piscivore species (CV1) reflects a similar mode of shape change to that recovered along PC1, namely an extension of the posterior edge of the operculum to create a bone that is more dorsally-broad, and triangular in shape. The ER~PC2 plot indicates that this broadening occurs in more elongate species, which have more negative PC2 scores and generally tend to be limnetic, feeding on fish or larger zooplankton. Conversely, deeper bodied species tend to be benthic, eating mainly algae, copepods and other small invertebrates, and have higher scores along PC2, reflecting a narrow operculum. In complement, results from the CVA based on feeding mode also clearly separate ram and suction feeders, which possess generally more broad opercles with a dorsally-flattened margin, from species that pick food from substrate and generally have a narrower bone.

Interspecific variation in operculum shape has previously been associated to a species’ position along the benthic-pelagic axis in the species flock of Antarctic notothenioids^29^. That study, however, also revealed a high level of phylogenetic structuring of shape space, a pattern not recovered among the LT cichlids. Beyond a number of studies that have identified high levels of variation in cichlid trophic apparatus^51^,^52^,^53^,^54^, a correspondence between aspects of craniofacial shape and feeding ecology has been previously shown for LT cichlids, particularly focusing on the evolution of Lower Pharyngeal Jaw (LPJ) shape^18^. Muschick and colleagues^18^ demonstrated that LPJ shape was highly similar among species with the same diet, and generally found a high level of convergence in LPJ as well as body shape. The latter was further demonstrated by comparisons between phylogenetic and morphological distances for species pairs, which clearly showed that LPJ and body shape was similar for species pairs that were phylogenetically distant from one another. In corroboration with the findings of Muschick *et al.*^18^, we find a relationship between operculum shape and feeding ecology, and evidence of convergence, though less marked than that detected for LPJ shape. Results of our pairwise distance-contrast plots indicated that more distantly related species were morphologically more similar than expected under neutral evolution (convergence) but also some species pairs showed divergence. The comparatively greater amount of convergence for LPJ shape in part reflects the unique functionality afforded by the pharyngeal jaw complex, which is recognized as an evolutionary key innovation in cichlids^53^, but also suggests there may be some difference in trophic trait rate diversification, which is further suggested by considering the disparity through time results. We find operculum shape and size disparity through time to be overall relatively constant with an increase towards present, in contrast to earlier findings for LPJ shape which showed a more marked elevation of disparity through time compared to neutral evolution, and also a continual decline in disparity to the present^18^. A direct explanation for these differences is not immediately obvious. They may reflect the differential importance of operculum shape to feeding, plus the potential role of the LPJ in courtship thus placing the trait under both natural and sexual selection^55^,^56^. While it is clear that operculum shape can evolve rapidly on a short time scale^37^, and there is evidence for strong directional selection along a specific axis of shape change that is not consistently biased by genetic architecture^39^, the uncovered shape changes, especially a broadening along the longitudinal axis of the bone, requires further investigation. Further, the DTT results also show no evidence for an ‘early burst’ scenario, which is consistent with a general scarcity of evidence for transient bursts of morphological evolution across a wide variety of animal clades^57^,^58^.

### Extracting general patterns on adaptive radiations in fishes

The correspondence of our recovered axes of shape variance in the operculum with those for other species flocks, and particularly for the extinct species flock of saurichthyid fishes, is encouraging in light of the quest for traits that may be studied across different radiations and in deep time. The importance of viewing adaptive radiation as a process, to assess axes of divergence through a global morphospace, rather than to elucidate patterns of diversity in a flock-specific morphospace is underscored by several studies that have uncovered convergence in axes of morphological diversification, for example across the cichlid radiations occurring in each of the East African Great Lakes^54^,^59^. One limiting aspect to this endeavor is that fewer characters may be examined in fossil species. To this end, study of the operculum, a bone that is commonly well-preserved, presents a promising source for continued research effort. Particularly, the discrimination of feeding mode and preference groups based on opercle shape could act as useful tools for inferring feeding ecology in fossil species, and may allow a more nuanced understanding of trophic niche exploitation in extinct species flocks.

Furthermore, that we find a relationship between operculum shape and body elongation is also encouraging for elucidating general patterns of morphological diversification in species flocks. Body elongation has been previously shown to be a major axis of body shape evolution in cichlids^19^ and in other fish groups, reflecting macrohabitat adaptation^44^,^53^,^60^. Recently, Maxwell and colleagues^47^ found a correlation between measures of opercle depth/length and body elongation among 10 saurichthyid species, suggesting that opercular depth may be constrained by a long, slender body and hypothesized that an axial length increase would necessitate an increased gill area to cope with increased metabolic requirements related to increased body mass in a more elongate form. This result is concordant with our findings, and shows that an interaction between elongation and opercle shape is present in at least one other species flock. This relationship, if uncovered as a general feature, may suggest that investigation of the operculum in fossils could provide insight into the evolution of elongation for specimens without fully preserved axial skeletons.

## Conclusions

We investigated patterns of operculum shape and size evolution in the cichlid fishes from Lake Tanganyika, and compare the patterns with those of an extinct species flock. Our results show that the major modes of operculum shape change among cichlids corresponds with those for other species flocks, and also for a sample of the Mesozoic saurichthyid fishes. Operculum shape patterns are found to be related to feeding, which may be used to gain insight into niche occupation and feeding ecology in fossil taxa, and to body elongation. We do not find evidence for an early burst of operculum trait evolution, instead recovering more support for a concentration of shape evolution towards present, and an increase in disparity around the time of the primary lacustrine radiation.

## Methods

### Study sample

**Figure 2:**
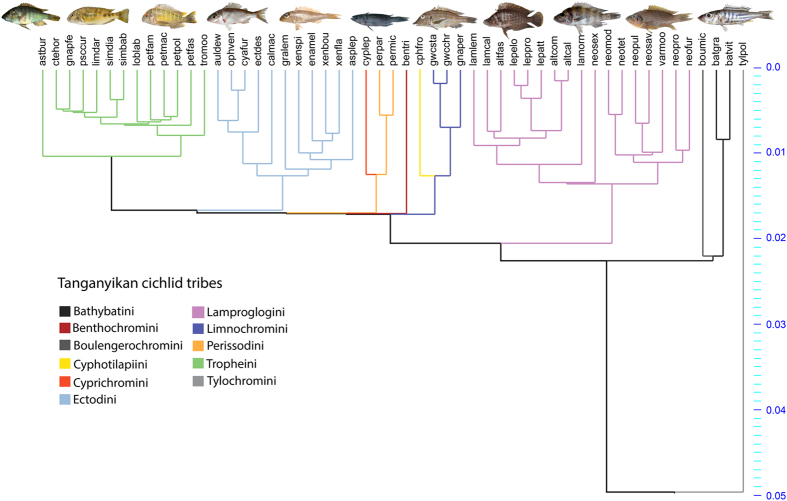
Phylogenetic relationships among the species studied here, pruned from the phylogeny of Muschick *et al.* (2012) which was based on two nuclear (*erdnrb1*, *phpt1*) and one mitochondrial (ND2) marker and the GTR+G model of molecular evolution. See Supplementary Table 1 for full details of species acronyms. Images shown are, from left to right: *Astatotilapia burtoni* (asbur), *Simochromis babaulti* (simbab), *Tropheus moori* (tromoo), *Cyathopharynx furcifer* (cyafur), *Xenotilapia spiloptera* (xenspi), *Perissodus microlepis* (permic), *Gnathochromis permaxillaris* (gnaper), *Altolamprologus calvus* (altcal), *Neolamprologus sexfasciatus* (neosex), *Neolamprologus pulcher* (neopul) and *Bathybates graueri* (batgra).

**Figure 3:**
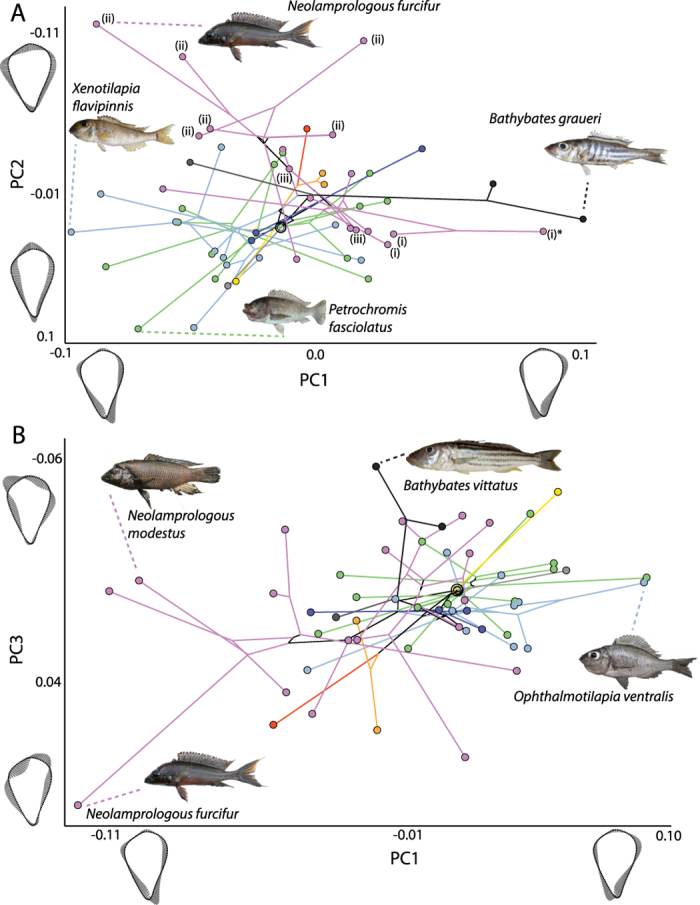
Phylomorphospace projections of cichlid relationships into operculum shape space, showing (A) PC1 vs. PC2 and (B) PC1 vs. PC3. Branches are colored by tribe (see Fig. 2), and the root is denoted by concentric ellipses. Patterns of outline shape change associated with each axis are illustrated using mean shape models and vector displacements. Labelled groups (i) *Altolamprologous*, (ii) *Neolamprologous*, (iii) *Lepidolamprologous* and taxon (i)* *Altolamprologous calva* are referred to in the text. Images of fish are not to scale.

**Figure 4:**
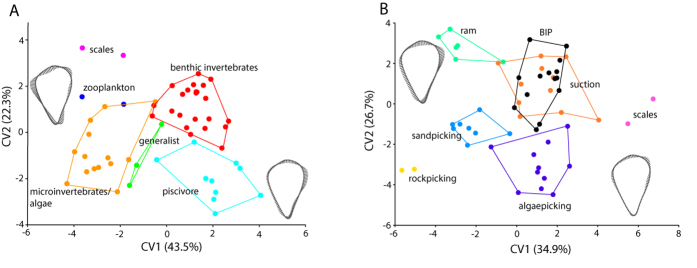
Results of Canonical Variates (CV) Analysis for operculum shape data, showing species mean values, grouped according to (A) feeding preference and (B) feeding mode categories.

**Figure 5:**
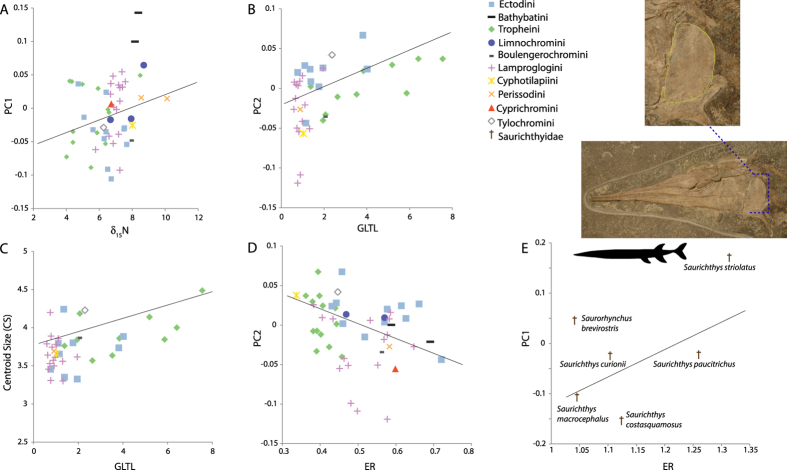
Phenotype-environment correlations for selected (significant) trait interactions, regression lines are produced using the Phylogenetic Generalized Least Squares (PGLS) model. Species mean values for centroid size and PC (axes 1 or 2) scores from a PCA of operculum shape data were plotted against mean values for: (**A**) δ_15_N, which is a proxy for trophic level wherein larger values reflect a higher trophic position; (**B**,**C**) gut length standardized by total length (GLTL); (**D**) Elongation Ratio (ER), and (**E**) Elongation Ratio for specimens belonging to the extinct species flock of saurichthyids (denoted by ϯ). A generalized sketch of the elongate body plan of saurichthyids is shown (modified from^27^), and inset a photograph of *S. macrocephalus* T4106 (Paläontologisches Institut und Museum, Zürich; photo: Rosi Roth). The dashed line indicates the position of the operculum, highlighted inset by a colored outline.

**Figure 6:**
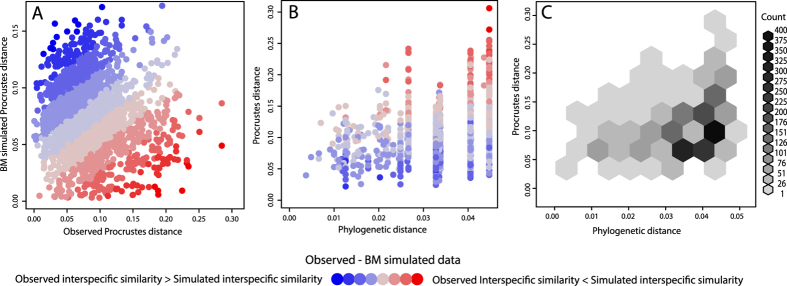
Pairwise distance-contrast plots, (A) colored using the difference between observed and simulated interspecific morphological distance, (B) showing the relationship between phylogenetic and morphological distance for all species pairwise comparisons data points, and for (C) binned values using N = 8 hexagonal bins. Interspecific morphological distances were simulated using Brownian motion to assess the relative similarity in shape between species pairs compared to that expected under neutral evolution on the given phylogeny.

**Figure 7:**
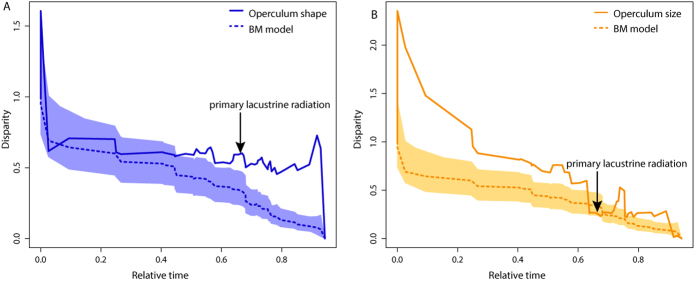
Disparity Through Time (DTT) plots for operculum shape (A) and centroid size (B) data. Mean values were used for each species, following the relationships depicted in Fig. 2. Disparity along the Y axis is the average subclade disparity divided by total clade disparity and is calculated at each internal node of the tree. The dotted lines represent values of trait disparity expected under Brownian motion by simulating operculum size and shape evolution 10000 times each across the tree. For relative time values 0.0 represents the root and 1.0 the tip of the phylogeny. Shaded areas on each plot indicate the 95% confidence interval for the simulations. The approximate timing of the primary lacustrine radiation, a synchronous diversification within several lineages that is thought to have coincided with the establishment of deep-water conditions in a clear lacustrine habitat^8^,^9^,^10^ is indicated.

The study sample comprised 416 specimens (54 species), representing 31 genera (of 53) and 11 of the 14 tribes present in the lake^10^ (Fig. 2). Additionally, we include data from 44 specimens, representing 5 species of saurichthyid fishes, previously collected by Wilson and colleagues^29^: *Saurichthys striolatus*, *S. costasquamosus*, *S. curionii*, *S. paucitrichus*, and *S. macrocephalus*. We collect new data for two specimens of *Saurorhynchus brevirostris*, housed at the Bayerische Staatssammlung für Paläontologie und Geologie München (Munich, Germany), and the Urweltmuseum Hauff (Holzmaden, Germany). These six species encapsulate the full range of body size variation within the clade, including both small-bodied *Saurichthys striolatus* (100–180 mm^48^), and several large-bodied species (e.g. *S. costasquamosus,* > 1 meter^41^), as well as spanning deposits from the Early Jurassic to the Late Triassic (Table 2^42^). Sampling was chosen to maximize usage of available data for body elongation^44^, and thereby enable direct comparison with cichlid data (see below) (Supplementary Table 1). Our dataset contains a considerable proportion of species present in LT (Fig. 2), including the most abundant ones that coexist in the southern basin of the lake^19^, and spans the majority of LT cichlid tribes. Ecological diversity is well represented in the sample, which includes epilithic algae grazers, scale eaters, fish hunters, invertebrate pickers and species that dwell in sandy, rocky or open water areas.

### Geometric morphometric data collection

Each specimen was photographed according to a standard procedure that has been used previously for geometric morphometric studies of the operculum^28^,^29^ and whole body shape^18^. For the cichlids, a Nikon D5000 digital camera mounted on a tripod, with the camera lens positioned parallel to the plane of the fish in lateral view, was used to capture the left side of head (see procedure described by Muschick and colleagues^18^), and for the saurichthyid specimens a similar protocol was performed followed by re-orientation of the image in Photoshop CS6 to correspond with life position (see^29^). Following the same approach as our previous studies^28^,^29^, the outline of each opercle was captured by 100 equi-distant semilandmarks collected using the software tpsDig^61^. This involved resampling the length of the outline clockwise, beginning at a homologous start point, defined by a type II^62^ landmark located at the maximum of curvature of the dorsal margin of the opercle (see Fig. 2 in^29^ for precise scheme, and^63^ for details on sampling simple closed curves). Coordinate points (x, y) were exported and centroid size was calculated for each specimen. Prior to analysis and ordination, landmarks were Procrustes superimposed to remove the effects of scale, translation and rotation.

Landmark data for all LT cichlid species were entered into Principal Component Analysis (PCA) to extract axes of maximum shape variance in the sample, and the broken stick model^64^ was used to assess significance of variance. A PCA was also conducted in Procrustes Form space for all cichlids, in which Procrustes shape coordinates plus the natural logarithm of centroid size are used as input^65^. We acknowledge that there are some concerns with the use of PC axes as proxies for phenotypic traits in the context of comparative methods (see^66^ for discussion), and our use of a comprehensive sampling of the ecomorphologcial diversity in LT cichlids helps to reduce any potential bias associated with the treatment of autocorrelated data from a PCA. Following Sidlauskas^67^, phylomorphospaces were constructed using PC axes and the plot tree 2D algorithm in the Rhetenor module of the software Mesquite^68^. For phylomorphospace ordinations, phylogenetic relationships for the 54 species in this study were derived from a pruned version of the phylogeny constructed by Muschick and colleagues^18^, which was based on sequences for one mitochondrial (ND2) and two nuclear (*ednrb1*, *phpt*) markers (Fig. 2).

Landmark data for all saurichthyid specimens (N = 44) were inputted into a separate PCA to extract the main axes of shape variance, and mean PC scores for six saurichthyid species were used in subsequent data plots.

### Exploration of operculum shape patterns associated with feeding preference and mode

Canonical variates analysis (CVA) of species’ mean landmark data was used to visualize the extent to which operculum shape reflected feeding preference and feeding mode groupings in LT cichlids. Data for feeding preference were collated from the literature^69^,^70^,^71^,^72^,^73^,^74^,^75^,^76^. Each species was assigned a feeding preference representing one of six categories: microinvertebrates/algae, zooplankton, benthic invertebrates, piscivore, scales, and ‘generalist’, which was used for opportunistic feeders (Supplementary Table 1). Each species was also assigned to one of seven feeding mode categories, these were: ram, sandpicking, rockpicking, scales, algaepicking, suction and benthic invertebrate picking (BIP). Procrustes ANOVAs were conducted on landmark data for all LT cichlid specimens to assess the effect of feeding preference and mode on operculum shape and size (e.g. 77).

### Correlation between operculum shape and ecological trait and niche data

Seven traits were used as covariates in this study: δ_13_C, δ_15_N, gill raker number on the ventral arch, gill raker number on the dorsal arch, average gill raker length, and gut length^19^, and elongation ratio (ER) (Colombo *et al.* in prep). All seven traits were available for LT cichlids, and ER was available for the fossil saurichthyid sample. Stable isotopes for δ_13_C and δ_15_N were used as proxies for specialization along the benthic-limnetic axis (macrohabitat) and tropic niche (microhabitat), respectively^78^. Features of the gill rakers, the bony processes that project from the gill arches, have been recently examined for LT cichlids, including number of gill rakers on the ventral arch (grnVa) and dorsal arch (grnDa), as well as mean gill raker length measured in millimeters (mean_rl) (see 19). Plasticity in intestinal length in response to quality of diet has previously been shown for LT cichlids; species that have low quality (nutrient poor) diets (e.g. algivores) have longer intestines to maximize the extraction of nutrients and energy from dietary material^76^. Gut length data (GLTL) were standardized against total body length for comparison across taxa. Elongation ratio (ER) is defined as the standard length of the body divided by its second largest major axis, which for the here measured cichlids refers to body depth (see Colombo *et al.* in prep). Elongation ratio (ER) data were taken from Maxwell and Wilson^44^ (therein referred to as ‘fineness ratio’) for saurichthyid species.

Using species mean values, interactions between operculum shape (PC1 and PC2) and form (PC1) space axes and centroid size in relation to the above seven traits were examined using Phylogenetic generalized least squares (PGLS) regression. PGLS takes phylogenetic relationships into account, assuming that the evolution of residual traits follows a neutral model (Brownian motion)^79^,^80^. PGLS was implemented in version 3.1.2 of R^81^ using the package *nlme*^82^(version 3.1–118). These analyses were conducted on a reduced data set for which all ecological variables were available (N = 38–49 species). PGLS regressions were also conducted for operculum shape (PC1 and PC2) and ER, using phylogenetic relationships taken from Maxwell *et al.* 43.

### Macroevolutionary model tests of operculum shape and size evolution

Several models were fit to the LT cichlid operculum shape data (axes PC1-PC3) and centroid size data, using the fitContinuous() function in the R package *Geiger*^83^(version 2.0.3). Model fit was assessed using sample-size corrected Akaike Information Criterion (AICc), and Akaike weight values were calculated to express proportional support for each model^84^. To enable direct comparison with a previous, comprehensive study of ecological and shape trait data in LT cichlids^19^, we fit Brownian motion (BM), Ornstein-Uhlenbeck (OU), and white noise (WN) models to evaluate the general process of operculum size and shape trait evolution. Under BM, trait evolution is simulated as a random walk through trait space, and phenotypic difference between sister taxa is expected to grow proportional to the sum of branch lengths between them. The OU model describes trait evolution under stabilizing selection, whereby there is attraction to a selective optimum, the strength of attraction to this selective optimum (i.e. the strength of selection) is measured using the alpha parameter. Under the WN model, equating to OU with an alpha of infinity, data are assumed to arise from a single normal distribution with no phylogenetically induced covariance among species values.

The time-dependence of trait evolution was assessed using Pagel’s δ model^85^ and the Early Burst (EB) model, also called the ACDC model (accelerating-decelerating^86^). Pagel’s δ model was used to evaluate whether changes in operculum trait data mainly occurred near the root (early) or tips (late) of the phylogeny. Values of <1 for δ indicate that branch lengths of the phylogeny are transformed to become increasingly shorter towards the tips and hence trait change occurred mainly along basal branches, whereas values of >1 for δ indicate trait evolution was more concentrated in younger subclades. The EB model measures, using the rate change parameter alpha, the acceleration or deceleration of evolution through time. Negative values of α reflect a rate deceleration in trait evolution whereas positive values indicate acceleration in trait evolution rate.

To quantify phylogenetic signal in operculum shape (axes PC1-PC3) and centroid size data, Blomberg’s *K* statistic^86^ was calculated using the R package *Picante*^87^ (version 1.6–2). Values of >1 for the *K* statistic indicate that close relatives are more similar in operculum traits than expected given the topology and branch lengths, whereas values of <1 indicate less phylogenetic signal than expected under a Brownian motion model^86^. Pagel’s λ, a branch length transformation model, was calculated to assess the extent to which the phylogeny predicts covariance in operculum shape and size for the species here examined^84^. Values of λ range from 0, reflecting a star phylogeny and no phylogenetic signal, to 1, which recovers the Brownian motion model.

Pairwise distance-contrast plots were constructed following a similar approach to Muschick and colleagues^18^ to assess whether differences in operculum shape were smaller between species pairs than were phylogenetic distances, which would indicate convergent evolution. *Tylochromis polylepis* (tylpol) was removed from the data set due to its large distance from other taxa^18^. Morphological and phylogenetic distances between species pairs were calculated and plotted against one another. Morphological distances were calculated by extracting a variance-covariance matrix of Procrustes distances between each species. A Phylogenetic distance matrix was extracted using the cophenetic() function in R. To compare the observed data with that expected under BM, which would predict a correlation between phylogenetic and morphological distance (divergence), shape data were simulated on the phylogeny. An evolutionary variance-covariance matrix was extracted for operculum shape data using the ratematrix() function in R^88^ using *Geiger*^83^(version 2.0.3). The function sim.char() was then used to simulate neutral trait evolution under BM. The simulated pairwise comparisons were then compared to the observed data by subtracting the simulated data from the observed data. This resulted in negative values when species were more similar in shape in the actual data than the data simulated given their phylogenetic distance, and positive values when species were more similar in the simulated data. We used this vector to color-code our plots, and additionally conducted a test for pairwise comparison between the observed data and the simulated data. We generated a 95% confidence interval for the simulated data using 1000 bootstrap replicates, and counted the number of species pairs in the observed data that had a smaller value than the lower 95% threshold value of the simulated data.

### Disparity through time analysis

To evaluate how operculum size and shape disparity changed through time, disparity through time (DTT) analyses were implemented in the R package *Geiger*^83^ (version 2.0.3) for centroid size and PC axes. Morphological Disparity Index (MDI) values were calculated to quantify overall difference in the observed trait disparity compared to that expected under Brownian motion by simulating operculum size and shape evolution 10,000 times across the tree. The function dttFullCIs() was used, following Slater *et al.*^89^ to create 95% confidence intervals on the simulations and to test whether the values for MDI differed significantly from the BM simulations. Default settings of nsmims = 10,000 were used to obtain a stable *P* value. To correct for tip over dispersion, MDI values were calculated over the first 90% of the phylogeny.

## Additional Information

**How to cite this article**: Wilson, L. A. B. *et al.* Evolution of opercle shape in cichlid fishes from Lake Tanganyika - adaptive trait interactions in extant and extinct species flocks. *Sci. Rep.* **5**, 16909; doi: 10.1038/srep16909 (2015).

## Supplementary Material

Supplementary Information

## Figures and Tables

**Figure 1:**
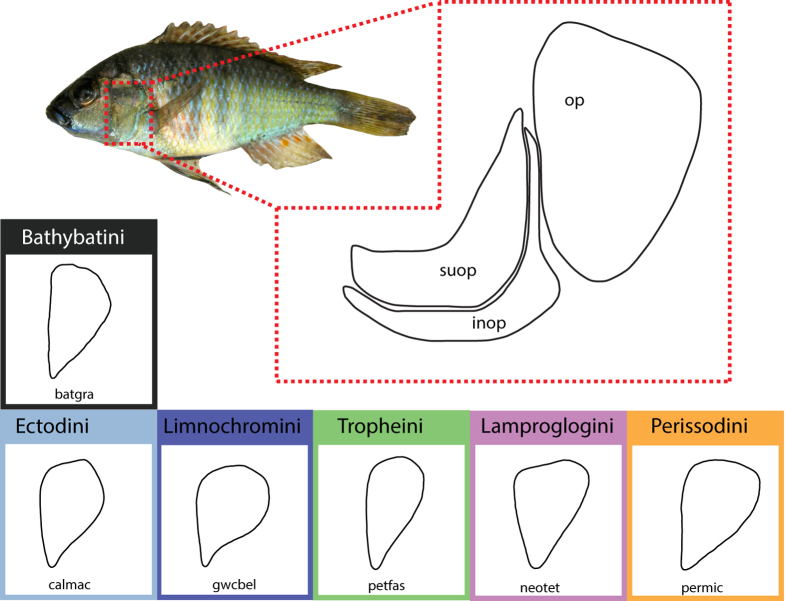
Photograph of *Astatotilapia burtoni* (astbur) showing the position of the operculum. Illustration of the operculum (op), and adjacent bones of the suboperculum (suop) and interoperculum (inop) are not to scale. Examples of operculum shape for several of the groups examined in this study are provided in the labelled, colored boxes. Species illustrated are: *Bathybates graueri* (batgra), *Callochromis macrops* (calmac), *Greenwoodochromis bellcrossi* (gwcbel), *Petrochromis famula* (petfas), *Neolamprologus tetracanthus* (neotet), and *Perissodus microlepis* (permic).

**Table 1:** Results of macroevolutionary models fit to axes of operculum shape (PC1-PC3) and centroid size data: Brownian Motion (BM), Ornstein-Uhlenbeck (OU), White noise (WN), Pagel’s delta (δ) and lambda (λ), Early Burst (EB). Akaike weight values were calculated using AICc (AIC corrected for sample size). Delta (d) AICc is calculated as the difference between the candidate model AICc and the AICc for the best fitting model (i.e. the one with the lowest AICc).

Variable	PC1				PC2				PC3				Centroid size			
Model	LogL	AICc	dAICc	Akaike Weight	LogL	AICc	dAICc	Akaike Weight	LogL	AICc	dAICc	Akaike Weight	LogL	AICc	dAICc	Akaike Weight
**BM**	77.53	−150.81	5.20	0.017	92.59	−180.93	4.97	0.021	105.25	−206.25	1.91	0.134	26.24	−48.22	1.66	0.106
**OU**	81.11	−155.71	0.29	0.193	96.09	−185.68	0.22	0.231	105.42	−204.33	3.82	0.051	28.10	−49.70	0.19	0.222
**WN**	80.13	−156.00	0.00	0.224	92.57	−180.90	4.99	0.021	105.27	−206.30	1.86	0.137	24.50	−44.75	5.13	0.019
δ	81.13	−155.74	0.26	0.197	96.13	−185.74	0.16	0.238	107.33	−208.16	0.00	0.347	28.20	−49.88	0.00	0.244
**EB**^1^	81.11	−155.71	0.29	0.194	96.10	−185.68	0.22	0.230	107.14	−207.77	0.39	0.286	28.10	−49.70	0.19	0.223
λ	—	−155.52	0.49	0.175	—	−185.90	0.00	0.258	—	−204.10	4.06	0.046	—	−49.36	0.53	0.187

^1^Alpha (α) values were 193.02 (PC1), 160.02 (PC2), 150.30 (PC3) and 106.94 (centroid size)

**Table 2:** Results of tests for phylogenetic signal in axes of operculum shape data (PC1-PC3) and centroid size data.

Variable	PC1	PC2	PC3	Centroid Size
Test				
Blomberg’s K Statistic	0.337	0.415	0.392	0.592
*P*	0.021	0.004	0.007	0.004
Pagel’s λ	0.746	0.777	0.945	0.851
