# Supplementary material for: Evolution of opercle shape in cichlid fishes from Lake Tanganyika - adaptive trait interactions in extant and extinct species flocks
Source: Sci Rep. 2015 Nov 20;5:16909. doi: 10.1038/srep16909 (PMC4653715; doi:10.1038/srep16909)

Supplementary Information

Evolution of opercle shape in cichlid fishes from Lake Tanganyika - adaptive trait interactions in extant and extinct species flocks

Laura A. B. Wilson, Marco Colombo, Marcelo R. Sánchez-Villagra, Walter Salzburger

Contents

Supplementary Table 1

Supplementary Fig. 1.

Supplementary Fig. 2.

Supplementary Table 1. List of species examined in this study.

Short name	Tribe	Species	N	Feeding preference	Feeding mode
altcal	Lamprologini	*Altolamprologus calvus*	3	benthic invertebrates	Suction
altcom	Lamprologini	*Altolamprologus compressiceps*	35	benthic invertebrates	Suction
altfas	Lamprologini	*Altolamprologus fasciatus*	20	benthic invertebrates	Suction
asplep	Ectodini	*Asprotilapia leptura*	9	microinvertebrates/ algae	Rockpicking
astbur	Tropheini	*Astatotilapia burtoni*	17	generalist	BIP
auldew	Ectodini	*Aulonocranus dewindtii*	31	microinvertebrates/ algae	BIP
batgra	Bathybatini	*Bathybates graueri*	4	piscivore	Ram
batvit	Bathybatini	*Bathybates vittatus*	3	piscivore	Ram
bentri	Benthochromini	*Benthochromis tricoti*	8	zooplankton	Suction
boumic	Boulengerochromini	*Boulengerochromis microlepis*	15	piscivore	Ram
calmac	Ectodini	*Callochromis macrops*	9	benthic invertebrates	Sandpicking
cphgib	Cyphotilapiini	*Cyphotilapia gibberosa*	13	piscivore	BIP
ctehor	Tropheini	*Ctenochromis horei*	9	generalist	BIP
cyafur	Ectodini	*Cyathopharynx furcifer*	23	microinvertebrates/ algae	Algaescraping
cyplep	Cyprichromini	*Cyprichromis leptosoma*	3	zooplankton	Suction
ectdes	Ectodini	*Ectodus descampsii*	3	generalist	Sandpicking
enamel	Ectodini	*Enantiopus melanogenys*	7	benthic invertebrates	Sandpicking
gnaper	Limnochromini	*Gnathochromis permaxillaris*	10	benthic invertebrates	Suction
gnapfe	Tropheini	*Gnathochromis pfefferi*	9	benthic invertebrates	Suction
gralem	Ectodini	*Grammatotria lemairii*	10	benthic invertebrates	Sandpicking
gwcbel	Limnochromini	*Greenwoodochromis bellcrossi*	6	piscivore	Suction
gwcchr	Limnochromini	*Greenwoodochromis christyi*	4	piscivore	BIP
intloo	Tropheini	*Interochromis loocki*	9	microinvertebrates/ algae	Algaescraping
lamcal	Lamprologini	*Lamprologus callipterus*	7	benthic invertebrates	BIP
lamlem	Lamprologini	*Lamprologus lemairii*	4	piscivore	Suction
lamorn	Lamprologini	*Lamprologus ornatipinnis*	4	benthic invertebrates	BIP
lepatt	Lamprologini	*Lepidiolamprologus attenuatus*	11	piscivore	Ram
lepelo	Lamprologini	*Lepidiolamprologus elongatus*	12	piscivore	Ram
leppro	Lamprologini	*Lepidolamprologus profundicola*	5	piscivore	Ram
limdar	Tropheini	*Limnotilapia dardenni*	15	microinvertebrates/ algae	Sandpicking
loblab	Tropheini	*Lobochilotes labiatus*	6	benthic invertebrates	Suction
neofur	Lamprologini	*Neolamprologus furcifer*	1	benthic invertebrates	BIP
neomod	Lamprologini	*Neolamprologus modestus*	7	benthic invertebrates	BIP
neopro	Lamprologini	*Neolamprologus prochilus*	3	benthic invertebrates	Suction
neopul	Lamprologini	*Neolamprologus pulcher*	8	benthic invertebrates	BIP
neosav	Lamprologini	*Neolamprologus savoryi*	2	benthic invertebrates	BIP
neosex	Lamprologini	*Neolamprologus sexfasciatus*	8	benthic invertebrates	BIP
neotet	Lamprologini	*Neolamprologus tetracanthus*	7	benthic invertebrates	BIP

ophven	Ectodini	*Ophthalmotilapia ventralis*	7	microinvertebrates/ algae	Algaescraping
permic	Perissodini	*Perissodus microlepis*	3	scales	Scales
perpar	Perissodini	*Perissodus paradoxus*	3	scales	Scales
petfam	Tropheini	*Petrochromis famula*	4	microinvertebrates/ algae	Algaescraping
petfas	Tropheini	*Petrochromis fasciolatus*	2	microinvertebrates/ algae	Algaescraping
petmac	Tropheini	*Petrochromis macrognathus*	1	microinvertebrates/ algae	Algaescraping
petpol	Tropheini	*Petrochromis polyodon*	4	microinvertebrates/ algae	Algaescraping
psccur	Tropheini	*Pseudosimochromis curvifrons*	4	microinvertebrates/ algae	Algaescraping
simbab	Tropheini	*Simochromis babaulti*	3	microinvertebrates/ algae	Algaescraping
simdia	Tropheini	*Simochromis diagramma*	2	microinvertebrates/ algae	Algaescraping
tromoo	Tropheini	*Tropheus moori*	10	microinvertebrates/ algae	Algaescraping
tylpol	Tylochromini	*Tylochromis polylepis*	2	benthic invertebrates	Sandpicking
varmoo	Lamprologini	*Variabilichromis moori*	2	benthic invertebrates	BIP
xenfla	Ectodini	*Xenotilapia flavipinnis*	2	benthic invertebrates	Sandpicking
xenbou	Ectodini	*Xenotilapia boulengeri*	4	benthic invertebrates	Sandpicking
xenspi	Ectodini	*Xenotilapia spiloptera*	3	benthic invertebrates	Rockpicking
		Saurorhynchus brevirostris	2		
		Saurichthys costasquamosus	4		
		Saurichthys curionii	15		
		Saurichthys macrocephalus	3		
		Saurichthys paucitrichus	1		
		Saurichthys striolatus	19		
**TOTAL**			**460**		

Supplementary Fig. 1. Phylomorphospace of PC1 and PC2 for form (shape + centroid size) space of opercular landmarks collected on specimens of Lake Tanganyikan cichlid fish.

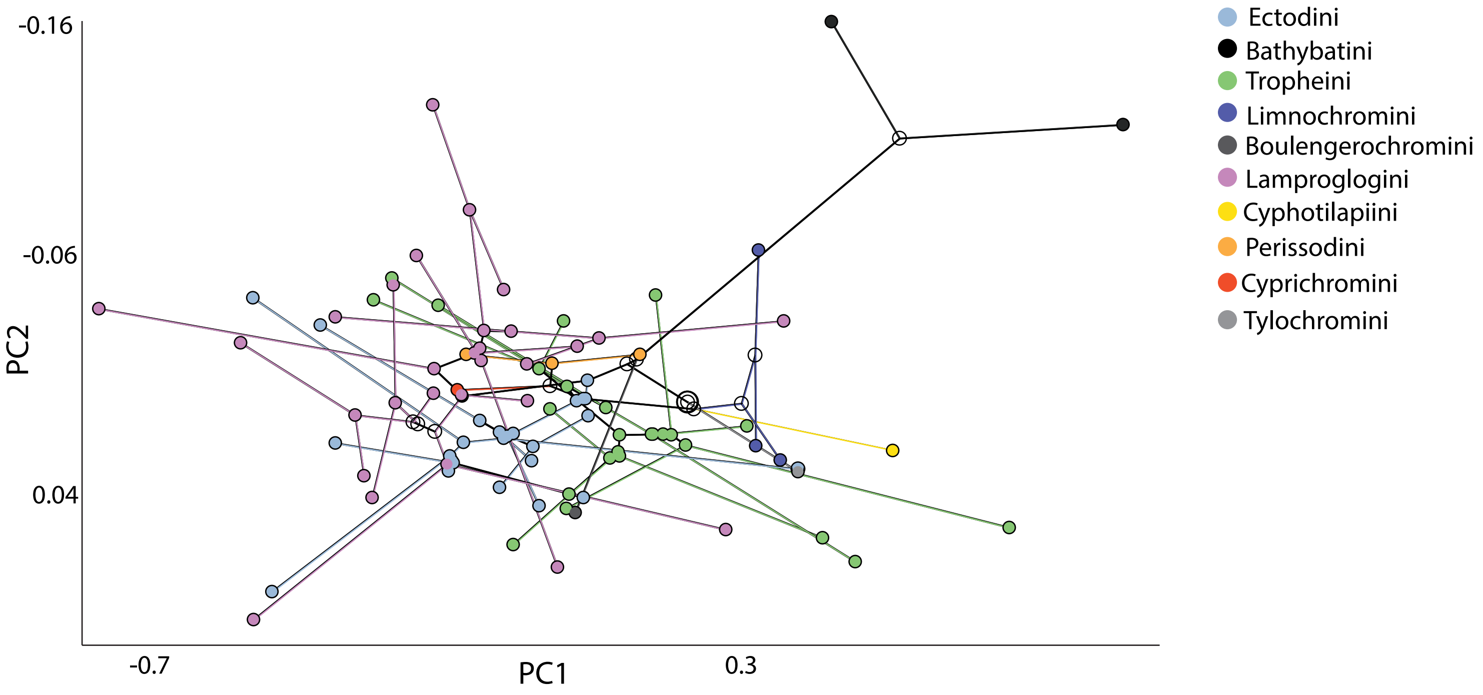

Supplementary Fig. 2. Ordination of PC1 (76.9%) and PC2 (8.7%) from PCA of opercular landmarks for members of the Saurichthyidae species flock.

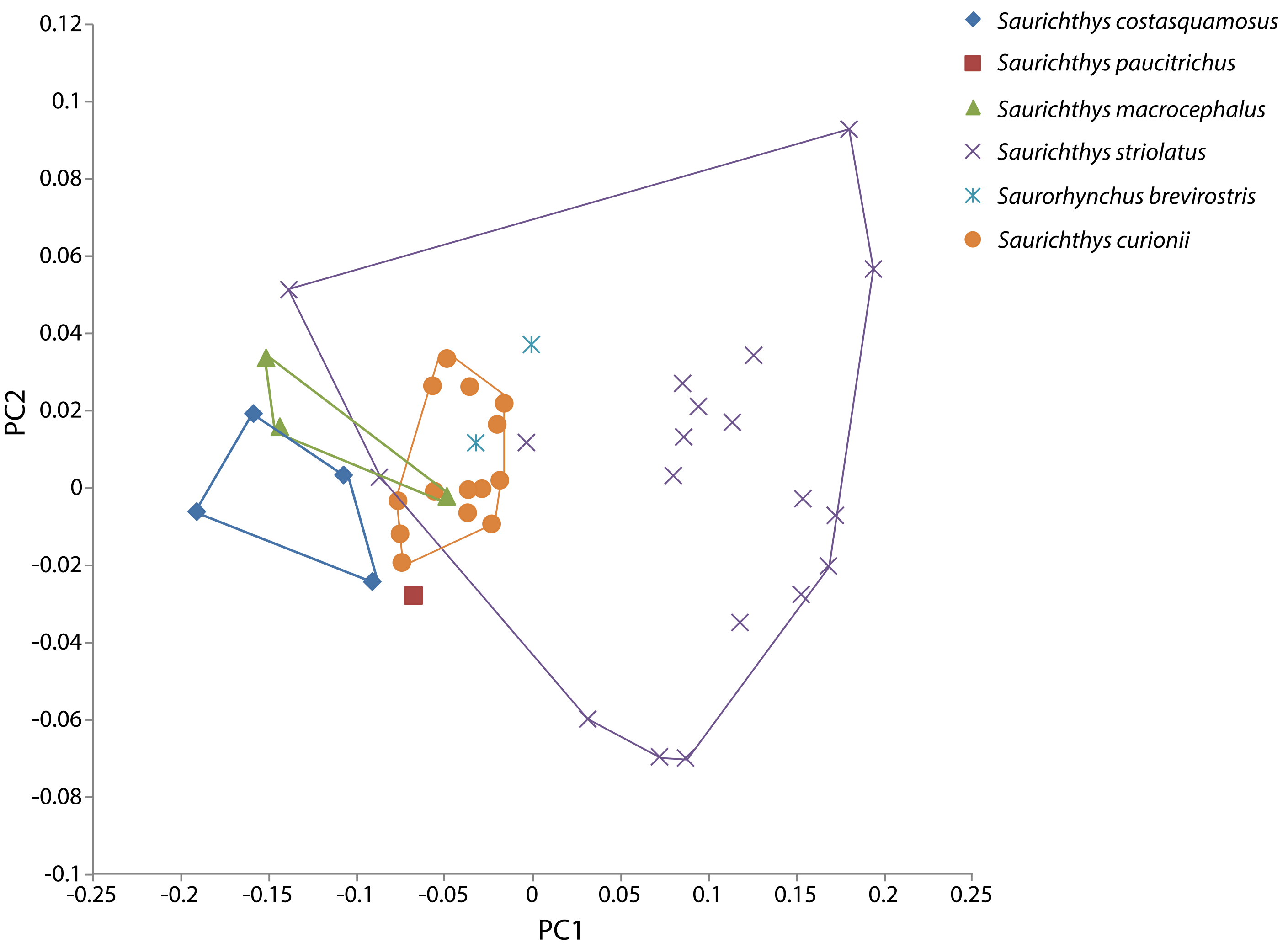

Supplement: Supplementary Information [file srep16909-s1.pdf]
